# Chinese herbal medicines and nutraceuticals inhibit *Pseudomonas aeruginosa* biofilm formation

**DOI:** 10.1099/acmi.0.000254

**Published:** 2021-08-17

**Authors:** Minami Hayashi, Hiroshi Kaneko, Tetsuya Yamada, Hideaki Ikoshi, Norihisa Noguchi, Hidemasa Nakaminami

**Affiliations:** ^1^​ Department of Clinical Microbiology, School of Pharmacy, Tokyo University of Pharmacy and Life Sciences, 1432-1 Horinouchi, Hachioji, Tokyo, Japan; ^2^​ Department of Traditional Chinese Medicine, School of Pharmacy, Tokyo University of Pharmacy and Life Sciences, 1432-1 Horinouchi, Hachioji, Tokyo, Japan

**Keywords:** biofilm, Chinese herbal medicine, nutraceutical, *Pseudomonas aeruginosa*

## Abstract

*
Pseudomonas aeruginosa* is a major biofilm-forming, opportunistic pathogen. Tolerance to antimicrobial agents due to biofilm formation may lead to the emergence of antimicrobial-resistant bacterial strains. Thus, adjunctive agents that can inhibit biofilm formation are necessary to enhance the therapeutic efficacy of antimicrobial agents. In this study, we evaluated the anti-biofilm formation activity of selected Chinese herbal medicines and nutraceuticals, which are commercially available in Japan. Among the eight agents evaluated for their potential to inhibit biofilm formation, Eiekikaryu S, Iribakuga and Hyakujunro significantly reduced *P. aeruginosa* biofilm formation (*P* <0.05) without inhibiting bacterial growth. Additionally, the expression of biofilm-associated genes (*rhlR*, *rhlA* and *lasB*) in *P. aeruginosa* was significantly suppressed by Eiekikaryu S, Iribakuga and Hyakujunro (*P* <0.001). Our findings indicate that some Chinese herbal medicines and nutraceuticals can be potential adjunctive agents for antimicrobial therapy against *P. aeruginosa *.

## Introduction

*Pseudomonas aeruginosa *, an environmental bacterium, exhibits low virulence and antimicrobial susceptibility and can readily acquire antimicrobial resistance [1]. It is a pathogen that causes opportunistic infections and has high biofilm-forming potential. Biofilm formation in *P. aeruginosa* is regulated by quorum sensing via autoinducers [2]. Biofilms consist of various extracellular polymeric substances, such as extracellular polysaccharides, DNA, proteins and lipids, and serve as a barrier that protects bacteria against antimicrobial agents [3]. Hence, biofilm-associated infections caused by *P. aeruginosa* are likely to become intractable. Tolerance to antimicrobial agents resulting from biofilm formation may lead to the emergence of antimicrobial-resistant bacterial strains. The therapeutic efficacy of antimicrobial agents can be enhanced by the co-administration of adjunctive agents with anti-biofilm formation activity [4].

Quorum-sensing mechanisms regulated by Rhl and Las contribute to *P. aeruginosa* biofilm formation [5]. In any quorum-sensing system, biofilm formation is accelerated by the production of autoinducers, elastase and rhamnolipids. These quorum-sensing systems are closely related, and the Las system positively regulates the Rhl system. They can serve as therapeutic targets for anti-biofilm formation agents. Hu *et al*. reported that herbal extracts can inhibit *P. aeruginosa* biofilm formation by inhibiting the transcription of biofilm-associated genes [6]. Furthermore, Wajima *et al*. reported that *Oldenlandia diffusa* extract, a Chinese herbal medicine, suppresses *Haemophilus influenzae* biofilm formation by inhibiting its quorum-sensing system [7].

The aim of this study was to explore the use of anti-biofilm formation agents against *P. aeruginosa *. Here, we evaluated the anti-biofilm formation activity of select Chinese herbal medicines and nutraceuticals, which are commercially available in Japan.

## Methods

### Bacterial strains, growth conditions and drugs

For this study, we chose *P. aeruginosa* PAO-1, a biofilm-forming strain [8]. The strain was cultivated on Tryptone Soya agar (Oxoid Ltd, Basingstoke, UK), Tryptone Soya broth (TSB; Oxoid), or Mueller–Hinton broth (MHB; Oxoid) at 35 °C. The Chinese herbal medicines and nutraceuticals used in this study were purchased from Iskra Industry Co., Ltd (Tokyo, Japan) (Table 1).

**Table 1.** Chinese herbal medicines and neutraceuticals used in this study

Category	Drug	Indication(s)	Composition(s)
Medicine	Eiekikaryu S	Weak constitution, fatigue	*Astragalus* root, *Atractylodes* rhizome, *Saposhnikovia* root and rhizome
	Ken-ikaryu S	Gastrointestinal weakness, diarrhoea	*Atractylodes* rhizome, *Poria* sclerotium, *Pinellia* tuber, *Amomum* seed, *Citrus unshiu* peel, *Glycyrrhiza*, *Saussurea costus*, *Codonopsis tangshen*
	Shimpikaryu	Anaemia, insomnia	*Astragalus* root, *Jujube* seed, *Atractylodes* rhizome, *Poria* sclerotium, *Longan* aril, *Polygala* root, *Angelica* root, *Glycyrrhiza*, *Saussurea costus*, *Codonopsis tangshen*
	Souryoukogikukaryu S	Hot flash, oedema, dizziness	*Rehmanniae radix*, *Corni fructus*, *Dioscoreae* rhizome, *Alismatis* tuber, *Pachyma hoelen*, dizziness *Moutan cortex*, *Chrysanthemi flos*, *Lycii fructus*
Nutraceutical	Iribakuga	Gynaecological disorder	Malt
	Shousansen	Dyspepsia, hangover	Hawthorn, malt, *Houttuynia cordata*, *Perilla*, *Garland chrysanthemum*, *Azuki* bean, *Apricot* kernel
	Shanseiyojin	Short attention span	American ginseng
	Hyakujunro	Dryness	*Lilium* bulb, *Glehnia* root and rhizome, Solomon’s seal

### Growth inhibition assay

A growth inhibition assay was performed as described in our previous study with some modifications [9]. Overnight cultures [4×10^3^ colony forming units (c.f.u.) ml^−1^) of the bacterial strain were inoculated into MHB in the absence or presence of 5, 10 and 20 mg ml^−1^ of the agents and incubated with shaking (200 r.p.m.) for 6 h. The cultures were spread onto Mueller–Hinton agar (MHA; Oxoid) at 0, 1, 2, 4 and 6 h post-incubation. After 24 h, the growth inhibitory concentration of the Chinese herbal medicines and nutraceuticals was determined by enumerating the c.f.u. present on the respective MHA plates. The results were calculated as the mean±standard deviation (sd), which was derived from at least three biological replicates.

### Biofilm formation inhibition assay

Biofilm formation was evaluated using the crystal violet assay as described in our previous study with some modifications [7]. Briefly, *P. aeruginosa* was cultured overnight in MHB and diluted 1 : 100 in fresh TSB containing 0.5 % glucose and incubated until its optical density (OD)_600_ reached 0.9. The culture was diluted to 1 : 100 in fresh TSB containing 0.5 % glucose. In a 96-well microtitre plate (AGC Techno Glass Co., Ltd, Tokyo, Japan), 200 µl of this suspension was added and incubated in the presence or absence of agents for 20 h at 37 °C with shaking (200 r.p.m.). Then, each well was washed twice with phosphate-buffered saline (PBS) to remove planktonic bacterial cells. The biofilms were stained for 10 min with 0.1 % crystal violet (FUJIFILM Wako Pure Chemical Corporation, Osaka, Japan) and washed twice with PBS. The remaining crystal violet was dissolved in 200 µl acetic acid (30 %), and the absorbance of each well was measured at 490 nm using a Multiskan GO (Thermo Fisher Scientific, MA, USA). The assay was performed in 24-well plates, and at least 3 biological replicates were performed. The results were calculated as the mean±sd.

### Preparation of bacterial RNA and quantitative reverse-transcription polymerase chain reaction (qRT-PCR)

Isolation of total RNA and qRT-PCR of *P. aeruginosa* was performed as described in our previous study with some modifications [9]. Total *P. aeruginosa* RNA was isolated using a Blood/Cultured Cell Total RNA Mini Kit (Favorgen Biotech Corp., Ping-Tung, Taiwan, ROC). Overnight cultures (4×10^3^ c.f.u. ml^−1^) of the tested strains were inoculated into MHB in the presence or absence of Chinese herbal medicines and nutraceuticals and incubated with shaking for 10 h. Real-time qRT-PCR was performed using the cDNA prepared by ReverTra Ace (TOYOBO Co., Ltd, Osaka, Tokyo). The primers designed for qRT-PCR assays are listed in Table S1 (available in the online version of this article) [5]. Real-time PCR was performed using the StepOne Real-Time PCR system (Thermo Fisher Scientific). All samples were analysed in triplicate, and expression levels were normalized against *rpoD* gene expression. The results were calculated as the mean±standard error of the mean (sem), which was derived from at least four biological replicates.

### Confocal microscopic analysis of the biofilm

Confocal microscopic analysis was performed as previously described, with some modifications [10]. In short, PAO-1 biofilms were generated in the wells of a CellCarrier-96 Ultra (PerkinElmer Inc., MA, USA) as described above. The biofilms were washed twice with PBS and fixed using 3.7 % formaldehyde (FUJIFILM Wako). The samples were then treated with a mixture of 10 % foetal bovine serum (FBS; GE Healthcare, IL, USA) and 0.5 % bovine serum albumin (Sigma-Aldrich Japan KK, Tokyo, Japan). Extracellular DNA, extracellular polysaccharides and lipids of biofilms were stained with 1 µg ml^−1^ 4′,6-diamidino-2-phenylindole, dihydrochloride (DAPI) (Dojindo Molecular Technologies, Inc., Tokyo, Japan), 2 µg ml^−1^ wheat germ agglutinin (WGA), Alexa Fluor 488 Conjugate (Thermo Fisher Scientific) and 10 µg ml^−1^ Nile Red (FUJIFILM Wako), respectively. Image J (http://imagej.nih.gov/ij/) and Comstat 2.1 (http://www.comstat.dk/) were used to calculate the biofilm thickness (µm) and biomass (µm^3^/µm^2^). The results were calculated as the mean±sem, which was derived from at least four biological replicates.

### Statistical analysis

The relative biofilm formation values, biofilm thickness and biomass were compared using Welch’s *t*-test. The relative levels of biofilm-associated gene transcription were compared using Scheffé’s test, followed by the Kruskal–Wallis test. Statistical significance was set at *P*<0.05.

## Results and discussion

### Inhibitory effect of Chinese herbal medicines and nutraceuticals on *P. aeruginosa* biofilm formation

The growth inhibitory concentrations of the Chinese herbal medicines and nutraceuticals against *P. aeruginosa* were assessed to determine the concentrations at which bacterial growth inhibition was not observed (Fig. S1). Most agents did not show any growth inhibition at the conventional dose (20 mg ml^−1^), excluding Shousansen (10 mg ml^−1^).

**Fig. 1.**
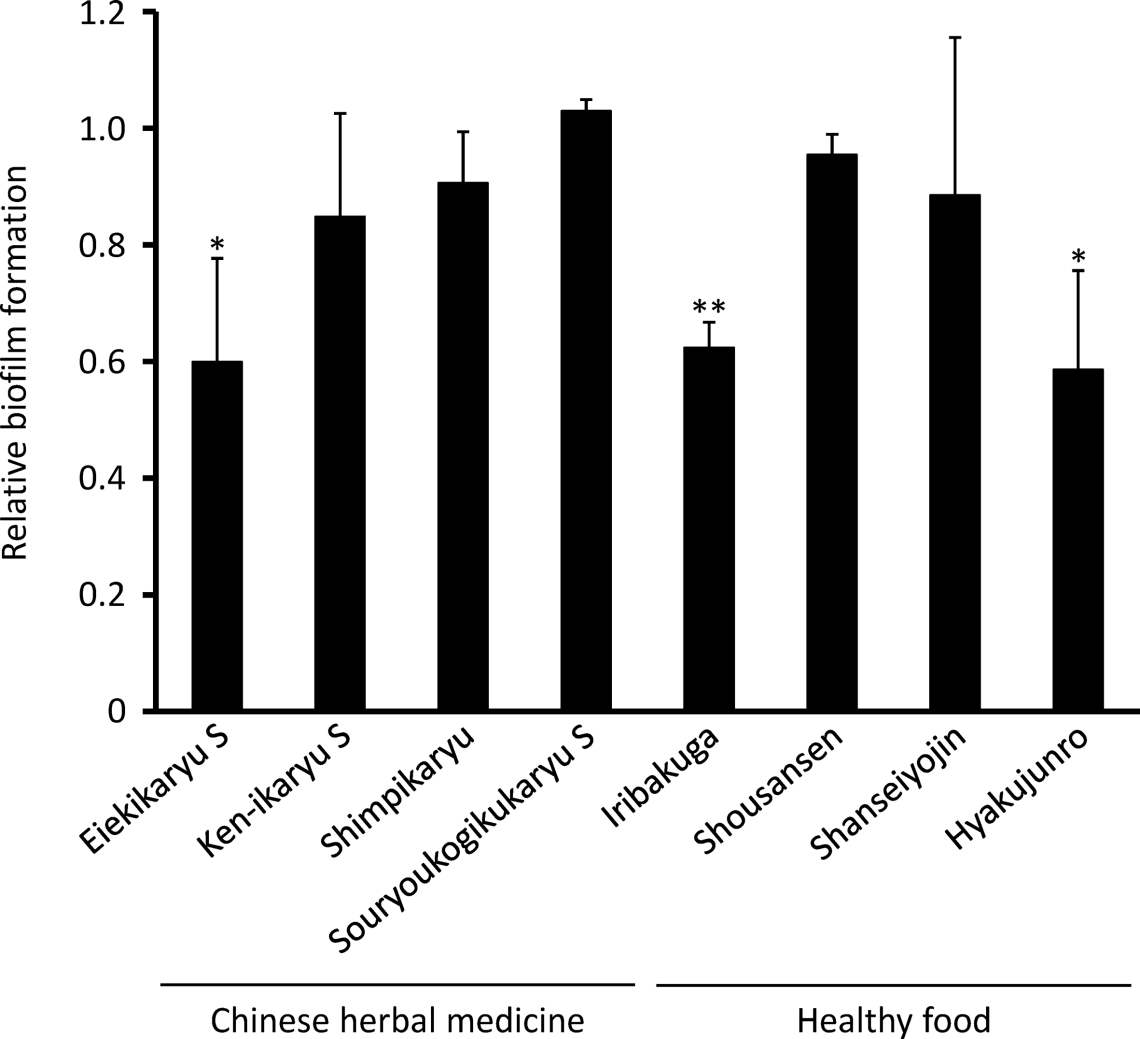
Inhibitory effect of Chinese herbal medicines and nutraceuticals on *P. aeruginosa* biofilm formation without any bacterial growth inhibition. *P <0.05 and **P <0.01 vs control.

**Fig. 2.**
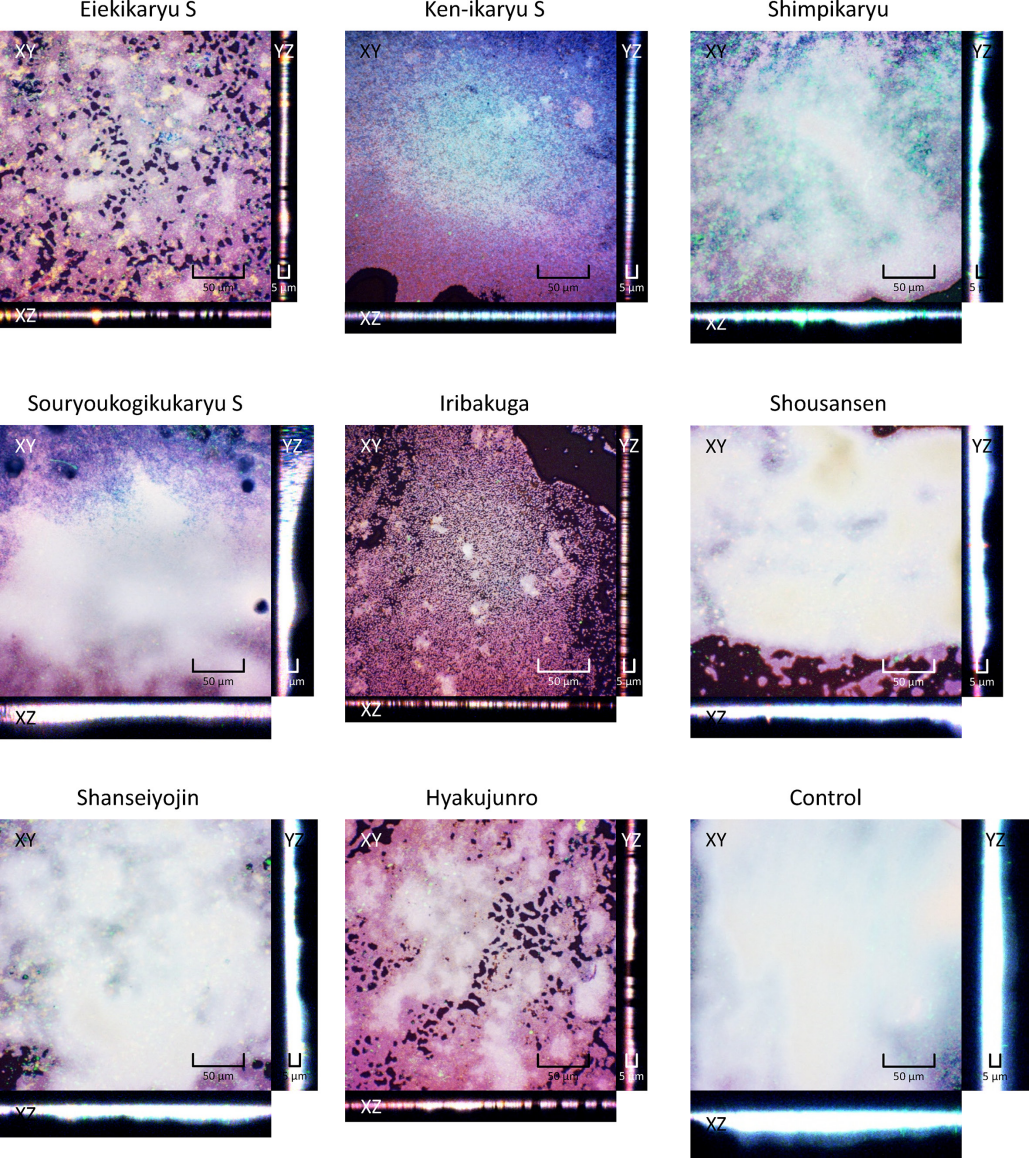
Confocal images of PAO-1-generated biofilms formed in the presence or absence of agents. Blue, extracellular DNA stained with DAPI; green, polysaccharides stained by WGA; red, lipids stained by Nile Red.

**Fig. 3.**
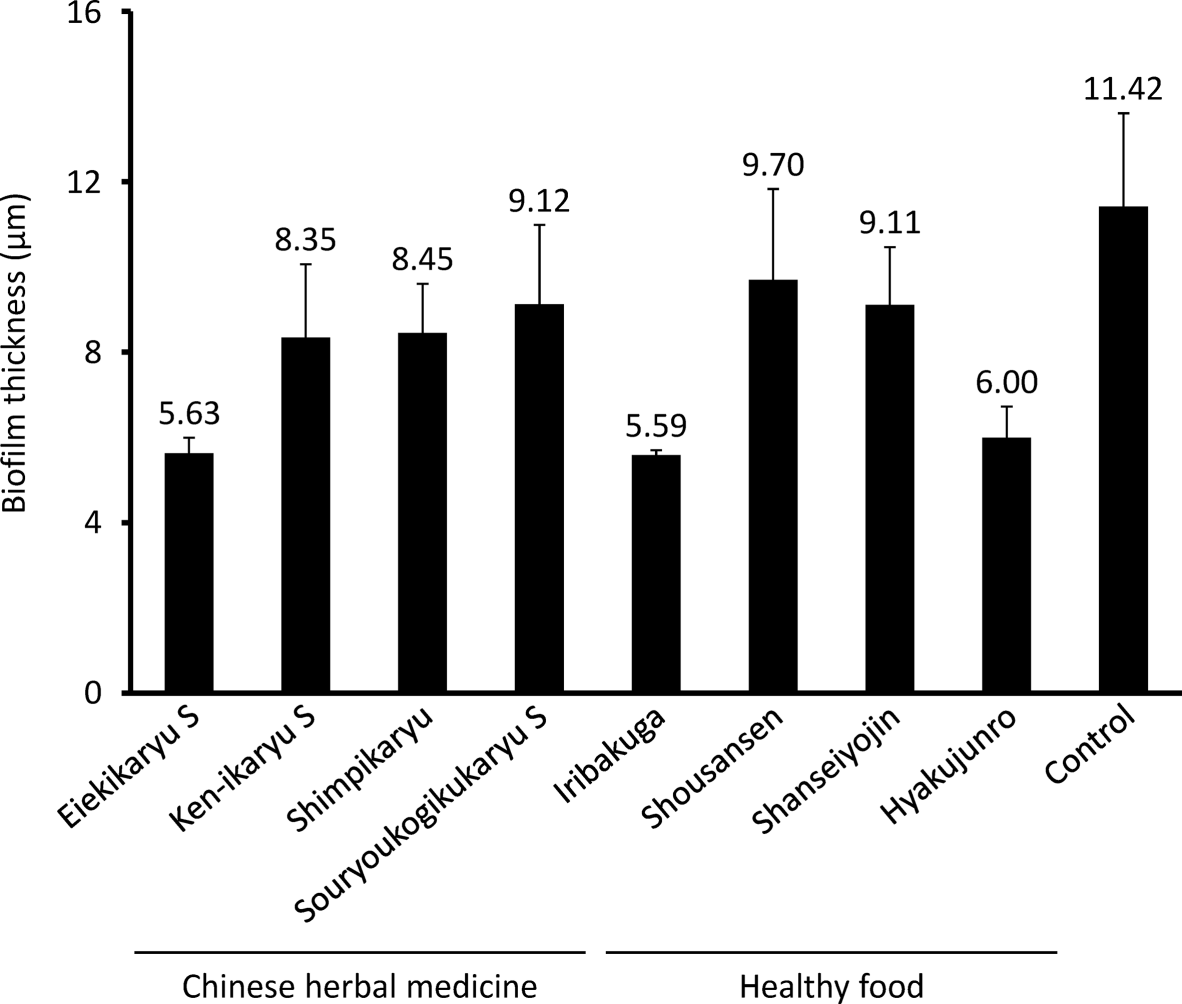
Thickness of PAO-1-generated biofilms formed in the presence or absence of agents.

Next, the anti-biofilm formation activities of the Chinese herbal medicines and nutraceuticals at the aforementioned concentrations were evaluated (Fig. 1). *P. aeruginosa* biofilm formation was significantly decreased by Eiekikaryu S (relative biofilm formation value=0.61), Iribakuga (relative biofilm formation value=0.62) and Hyakujunro (relative biofilm formation value=0.59) (*P* <0.05). The morphological and compositional impact of Chinese herbal medicines and nutraceuticals on *P. aeruginosa *-generated biofilms was analysed from fluorescence intensity distribution using confocal laser microscopy (Figs 2 and 3). Eiekikaryu S (biofilm thickness=5.63 µm), Iribakuga (biofilm thickness=5.59 µm) and Hyakujunro (biofilm thickness=6.00 µm) substantially changed the thickness and markedly reduced the occupied area of the biofilm. When the residual rates of biofilm components were determined, the extracellular DNA, polysaccharides and lipids of the biofilms were found to be significantly reduced by Eiekikaryu S, Ken-ikaryu S, Iribakuga and Hyakujunro compared to those in the control (*P*<0.05) (Table 2). These data show that Eiekikaryu S, Iribakuga and Hyakujunro significantly reduced *P. aeruginosa* biofilm formation without inhibiting bacterial growth.

**Table 2.** Total content of biofilm components

Chinese herbal medicine/ Healthy food	Relative biomass ±sem (%)		
	DNA	Polysaccharides	Lipids
Eiekikaryu S	45.4±7.5**	50.4±9.6**	54.9±7.2*
Ken-ikaryu S	62.3±3.0**	64.7±1.4**	66.7±2.8**
Shimpikaryu	75.8±14.1	77.8±14.0	74.9±10.5
Souryoukogikukaryu S	69.7±15.5	68.4±12.3	68.1±9.3
Iribakuga	43.7±8.3**	49.2±11.5*	51.3±7.4*
Shousansen	79.2±14.0	79.6±12.4	80.7±9.5
Shanseiyojin	80.1±12.9	78.3±8.1	76.0±5.3
Hyakujunro	45.6±3.2**	47.6±4.2**	53.8±3.0**
Control	100.0±0.0	100.0±0.0	100.0±0.0

*P <0.05, **P <0.01 vs control.

### Influence of Chinese herbal medicines and nutraceuticals on the expression of biofilm-associated genes in *P. aeruginosa*

**Fig. 4.**
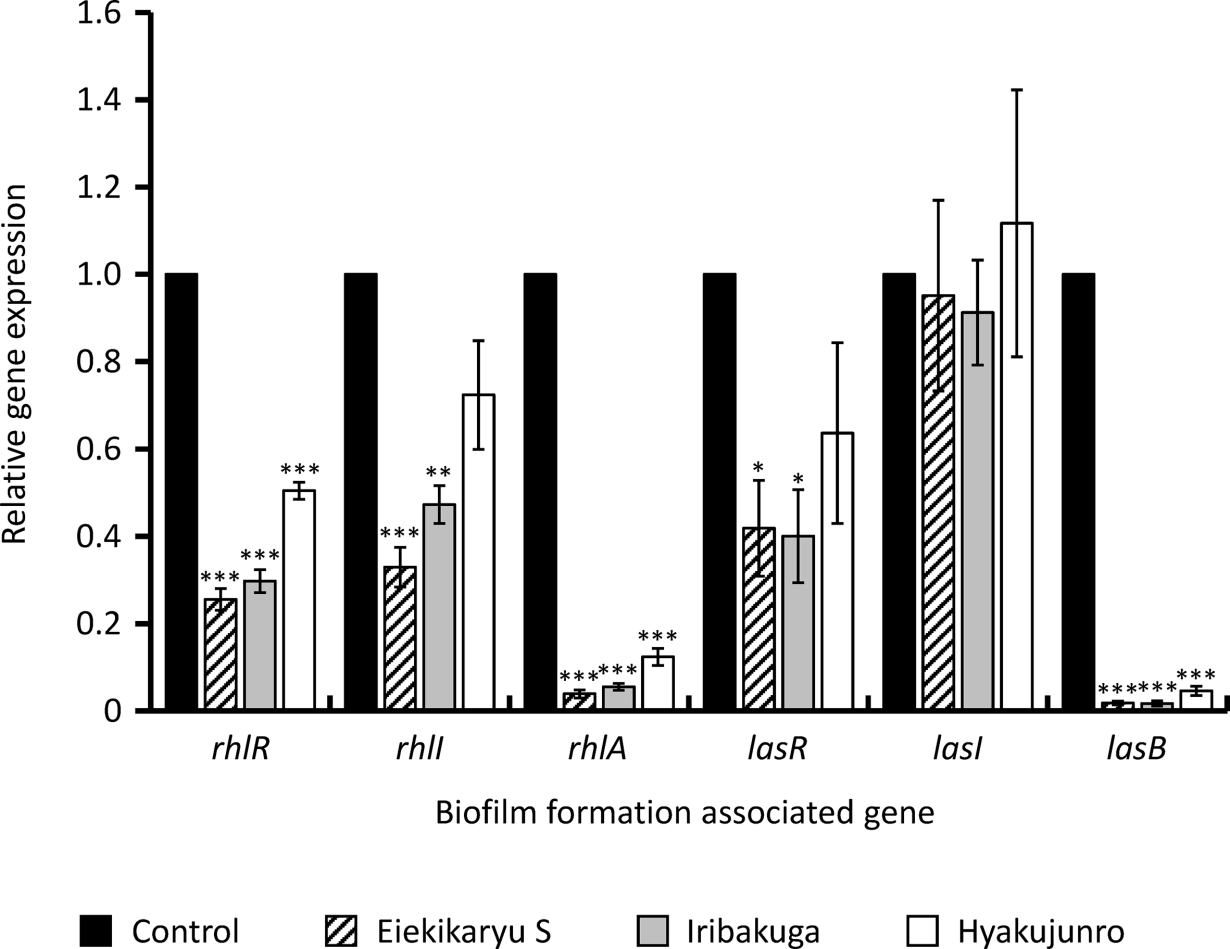
Expression of biofilm-associated genes of *P. aeruginosa* in the presence or absence of 20 mg ml^−1^ of Chinese herbal medicines or nutraceuticals. *P <0.05, **P <0.01 and ***P <0.001 vs control.

**Fig. 5.**
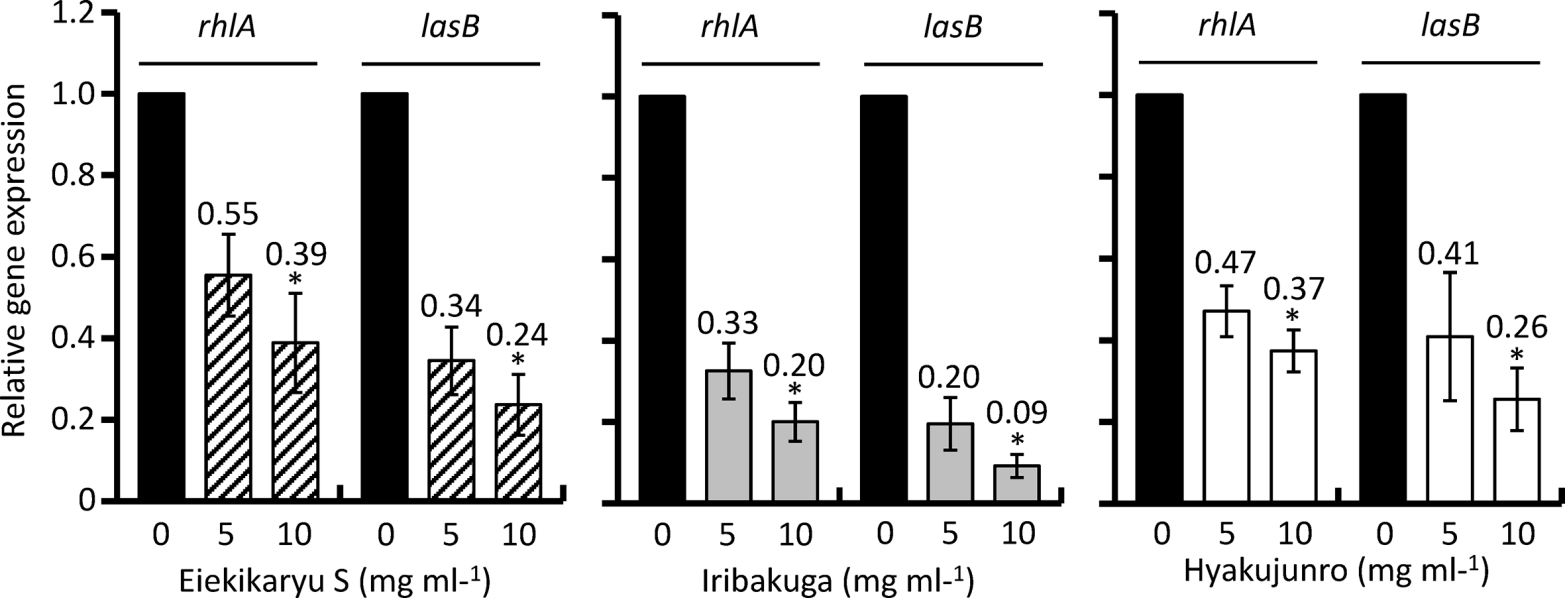
Suppression of gene expression of biofilm-associated genes of *P. aeruginosa* by Chinese herbal medicines and nutraceuticals in a dose-dependent manner. The values indicate relative gene expression vs control. *P <0.05 vs control.

To investigate the influence of Eiekikaryu S, Iribakuga and Hyakujunro on the expression of biofilm-associated genes, real-time qRT-PCR was conducted (Fig. 4). The expression of Rhl genes (*rhlR* and *rhlA*) and Las gene (*lasB*) was significantly suppressed by Eiekikaryu S (relative expression values=0.26, 0.04 and 0.02, respectively), Iribakuga (relative expression values=0.30, 0.06 and 0.02, respectively) and Hyakujunro (relative expression values=0.51, 0.12 and 0.05, respectively) (*P*<0.001). In particular, *rhlA* and *lasB* expression was notably suppressed in a concentration-dependent manner (Fig. 5).

These data indicate that the mechanism of the anti-biofilm formation activity was the downregulated expression of biofilm-associated genes. Inhibition of biofilm formation can support the penetration of antimicrobial agents into biofilm-embedded bacteria, thereby enhancing their therapeutic efficacy. Therefore, anti-biofilm agents may be potential adjunctive agents for antimicrobial therapy. No similarities were found in the herbal components of Eiekikaryu S, Iribakuga and Hyakujunro (Table 1). There is a very clear effect at the phenotypic and transcriptional levels, albeit not in a clinical isolate. However, further studies are required to elucidate the mechanism underlying this phenotypic response and establish the use of these Chinese herbal medicines and nutraceuticals as anti-biofilm agents in humans.

## Conclusion

Our findings showed that Eiekikaryu S, Iribakuga and Hyakujunro inhibited *P. aeruginosa* biofilm formation by suppressing the expression of biofilm-associated genes. Therefore, Chinese herbal medicines and nutraceuticals can be potential adjunctive agents for antimicrobial therapy against biofilm-forming *P. aeruginosa* infections.

## Supplementary Data

Supplementary material 1
