# Supplementary material for: Chinese herbal medicines and nutraceuticals inhibit Pseudomonas aeruginosa biofilm formation
Source: Access Microbiol. 2021 Aug 17;3(8):000254. doi: 10.1099/acmi.0.000254 (PMC8650844; doi:10.1099/acmi.0.000254)
Supplement: Supplementary material 1 [file acmi-3-0254-s001.pdf]

Table S1. Primers used in this study

Target gene	Direction	Sequence (5’ to 3’)	Tm (°C)	Product size (bp)	Reference
rpoD	Forward	ACAAGATCCGCAAGGTACTGAAG	58.7	87	[7]
	Reverse	CGCCCAGGTGCGAATC	54.0		
rhlR	Forward	AACGCGAGATCCTGCAATG	56.1	105	[7]
	Reverse	GCGCGTCGAACTTCTTCTG	58.2		
rhlI	Forward	GCAGCTGGCGATGAAGATATTC	58.6	108	[7]
	Reverse	CGAACGAAATAGCGCTCCAT	56.3		
rhlA	Forward	GGCGATCGGCCATCTG	54.0	69	[7]
	Reverse	AGCGAAGCCATGTGCTGAT	56.1		
lasR	Forward	GACCAGTTGGGAGATATCGGTTA	58.7	77	[7]
	Reverse	TCCGCCGAATATTTCCCATA	54.3		
lasI	Forward	GCCCCTACATGCTGAAGAACA	58.5	62	[7]
	Reverse	CGAGCAAGGCGCTTCCT	56.0		
lasB	Forward	CGACAACGCGTCGCAGTA	58.1	54	[7]
	Reverse	AGGTAGAACGCACGGTTGTACA	58.6		

Fig. S1

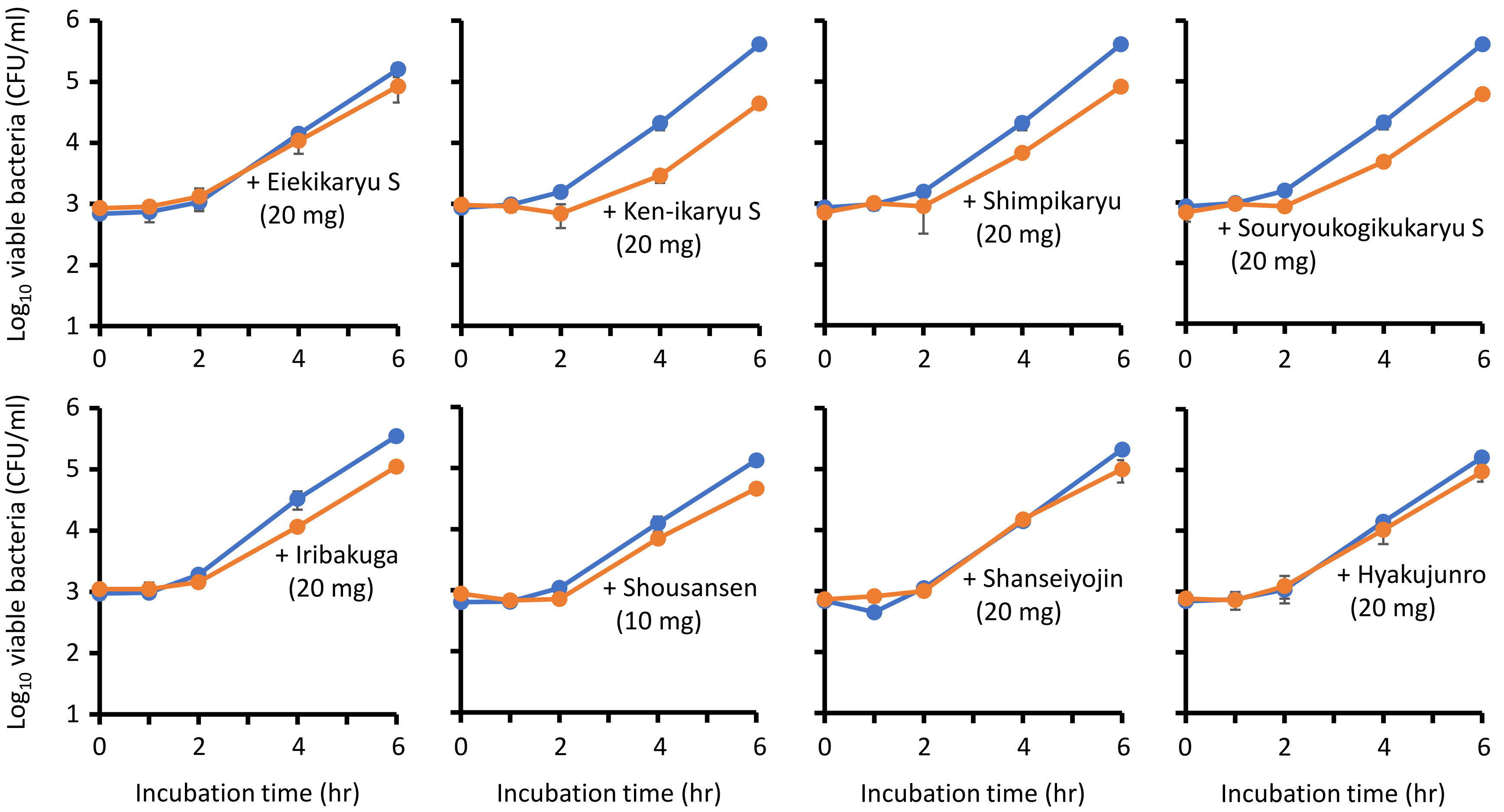

Fig. S1 Growth inhibition effect of Chinese herbal medicines and healthy foods against *P. aeruginosa*.
